# Content Analysis of the Free COVID-19 Telephone Consultations Available during the First Wave of the Pandemic in Japan

**DOI:** 10.3390/healthcare9111593

**Published:** 2021-11-20

**Authors:** Kyoko Yoshioka-Maeda, Yuka Sumikawa, Noriha Tanaka, Chikako Honda, Riho Iwasaki-Motegi, Noriko Yamamoto-Mitani

**Affiliations:** 1 Department of Health Promotion, National Institute of Public Health, Saitama 351-0197, Japan; 2 Department of Community Health Nursing, Division of Health Sciences and Nursing, Graduate School of Medicine, The University of Tokyo, Tokyo 113-0033, Japan; sumi-tky@g.ecc.u-tokyo.ac.jp (Y.S.); tanaka-noriha060@g.ecc.u-tokyo.ac.jp (N.T.); hchika-tky@g.ecc.u-tokyo.ac.jp (C.H.); riwasaki-tky@g.ecc.u-tokyo.ac.jp (R.I.-M.); noriko-tky@g.ecc.u-tokyo.ac.jp (N.Y.-M.)

**Keywords:** COVID-19, public health nurse, telephone consultation

## Abstract

This cross-sectional study aimed to (1) describe the unclassified contents of telephone consultation services provided by a public health center during the first wave of COVID-19 in Japan and (2) examine whether the contents required assistance from public health nurses (PHNs). We analyzed a total of 207 calls in which the purpose of the call was unclassified into pre-set categories. PHNs transcribed the exact text of the consultation conversations recorded from 25 March to 20 April 2020 in City A. Approximately half of the calls were from residents. Seven categories were extracted through a qualitative content analysis. The most common topic was infection control measures, where the presence of COVID-19 infection was assumed (*n* = 62); the second most common was extreme anxiety and fear of infection (*n* = 50). Questions about the COVID-19 response system (*n* = 30), discrimination and misunderstandings about COVID-19 (*n* = 24), and response measures for COVID-19 outbreaks within organizations (*n* = 18) were also included. The unclassified consultations included various topics, several of which required the expertise of a PHN. Each local government should consider sharing and task-shifting telephone consultation services among PHNs and other staff to reduce their burden and allow them to concentrate on conducting infection control more effectively.

## 1. Introduction

In the context of the coronavirus disease 2019 (COVID-19) pandemic, which has already claimed more than 2 million lives, preventing the further spread of infection is critical to saving lives [1]. Many countries enacted strict lockdowns as infection control measures to reduce interaction and maintain social distancing [2,3].

In the United States of America, the total number of cumulative deaths out of 56,589,190 COVID-19 cases was 1,368,633, as of 4 April 2020 [4]. In contrast, the total number of deaths in Japan was 84 on the same day from 3507 COVID-19 cases [5]. Due to fewer COVID-19 cases occurring in Japan than in other countries, the Japanese government did not mandate strict lockdowns and people voluntarily stayed at home to maintain social distancing. During the first wave of the COVID-19 pandemic, the first state of emergency nationwide [6] successfully decreased the rate of new COVID-19 cases occurring during April–May 2020.

Based on the Japanese Infectious Disease Prevention Act [7], public health centers (PHCs) are responsible for infectious disease control, and public health nurses (PHNs) have many crucial roles related to disease prevention. Their role has been particularly relevant in the COVID-19 pandemic. For example, they are responsible for conducting epidemiological surveys, coordinating each hospitalization case, monitoring the health condition of patients’ contacts, and providing telephone consultations to people in the community. However, due to the limited number of intensive care unit (ICU) beds and the maximum capacity for processing polymerized chain reaction (PCR) tests being reached [8], PHNs were only able to coordinate PCR testing for people who were in close contact with COVID-19 patients and those recommended to visit their designated hospital [9]. Furthermore, the progress made by administrative reform has diminished, and the number of PHCs in Japan has dropped by 60% since the 1990s [10], leaving the remaining PHCs at risk of dysfunction during the COVID-19 pandemic. The Centers for Disease Control and Prevention recommended that sharing and shifting tasks is crucial in providing essential healthcare services in the community [11]. To maximize PHNs’ time and expertise for the practice of infection control, each PHC needs to reduce the workload of PHNs through sharing and shifting their tasks with other staff members [12].

The national government of Japan has also started a free telephone consultation service for each PHC to prevent the spread of COVID-19 infection. However, the practice of telemedicine that has been promoted in clinical settings has not been fully advanced in PHC [13,14,15]. The PHC telephone consultation service aims to identify high-risk contacts, such as returnees from abroad, people suspected of having contact with patients confirmed to have COVID-19, and people who have fevers and respiratory symptoms, to advise them to visit their designated hospital for PCR testing [16]. Meanwhile, each day, the PHC staff face a rapidly increasing number of calls, such as calls about infection control practices and community members sharing their opinions and complaints regarding COVID-19 [17]. Thus, the telephone consultation service was not initially developed to field all the questions and concerns of the general public.

A previous study on this topic was conducted to reveal patterns in the content of the telephone consultations provided to people in the community [17]. The authors were not able to adequately analyze 18% of the consultations, which were not categorized into pre-set categories. However, the analysis of unclassified consultations is crucial to promote the task shifting and task sharing of PHCs with other staff members based on evidence in preparation for the next global pandemic. Therefore, the as-yet unclassified contents of the PHC telephone consultations need to be analyzed. In this study, we aimed to (1) analyze the unclassified content of PHC’s telephone consultations during the first wave of the COVID-19 pandemic and (2) examine which, if any, required consultations with PHNs and which could be managed by other staff members.

## 2. Materials and Methods

### 2.1. Design and Setting

A cross-sectional study was conducted to identify the characteristics of suspected COVID-19 cases throughout the whole community. The study sample comprised all the residents of City A with suspected symptoms of COVID-19 who had consulted the Coronavirus Telephone Consultation Center between 25 March and 20 April 2020. City A is a residential area in the Tokyo metropolis. Out of the population of 230,000, 19.2% of this area are aged 65 years and over [18].

In response to the expansion of COVID-19, the PHC in City A opened a free COVID-19 teleconsultation service for residents in February 2020. PHNs in charge of infection prevention and control at PHC in City A reviewed and summarized the contents of teleconsultation sheets and kept records of the content. Data gathered included the date of consultation, the attributes of the consultation (e.g., the caller’s gender and age, the time of the call, the length of time required), the contents of the consultation (including the content of pre-set categories and unclassified “other” content), and the consultant’s recommendations [17]. In the case of “other consultations”, PHNs recorded the text data of the consultation as spoken by the telephone consultant.

### 2.2. Ethical Considerations

This study was conducted following the principles of the Declaration of Helsinki. Ethical approval for this study was obtained from the Ethical Review Committee of the University of Tokyo, Japan (No. 2020138NI). Data were protected by storage in a locked cabinet, a password-locked spreadsheet, and a computer that was not connected to the Internet. To preserve the anonymity of the consulters (callers), we identified them only by the chronological order ID number assigned to each call.

### 2.3. Data Analysis

The research team analyzed the responses in the free-text field provided for the consultations classified as “other” using a qualitative content analysis approach that focused on the manifest content of the data [19]. Content analysis is a scientific process that allows us to gain new insight regarding particular situations and actions by analyzing large amounts of text. This type of analysis is appropriate for use on topics of a sensitive nature [20]. To ensure the reliability of the coding, the researchers conducting the data analysis all had the same professional background [20]. First, three researchers who had working experience as PHNs read and re-read the text data of the unclassified consultation conversation to gain an understanding of what the callers were talking about. The researchers divided the text into sentences based on their meanings using Microsoft Excel. Second, the researchers summarized each sentence unit using a code and sorted them based on the questions—e.g., “Why did the consultant want to ask this question?” and “Why did the consultant give this opinion?” Third, we labeled the subcategories and categories reflecting the classification according to the commonality of the sematic content. Then, we counted the total number of occurrences of each subcategory and category. We developed a schematic model based on whether the PHNs should have responded to calls in each category. We asked experts in qualitative research methods to ensure the validity of the results. Based on their opinions, we revised the names of the categories and the schematic model.

## 3. Results

### 3.1. Characteristics of Consultation Cases

A total of 188 consulters whose concerns had been among the 207 categorized as “other consultations” in the previous study were analyzed [17]. Approximately half of the unclassified concerns were from individual residents, and a quarter of the calls—more than the amount from any other age group—were from people in their 30s (see Table 1). The mean duration of each consultation was 7.0 min (SD = 4.6).

### 3.2. Category of “Other” Consultations

Seven categories and 24 subcategories were extracted during the qualitative content analysis (Table 2). Direct quotations from the text data of each consultation are provided within the quotation marks.

#### 3.2.1. Assumed COVID-19 Infection and Consulting about Infection Control

Even though the callers were not infected with COVID-19, they asked the PHNs how to respond to an outbreak of COVID-19 in their area or coming into contact with someone with COVID-19 as well as what measures to take in these cases. For example, this category included communicating with organizational members if a COVID-19 outbreak occurred within their organization, and the callers sought the learn the criteria concerning restricting and self-isolating after coming into contact with a COVID-19 patient. Additionally, they asked how to continue providing care when a caregiver was infected with COVID-19 or was unable to provide care.

“*What should we do if we have a positive case of COVID-19 in our office in the future? I would like to know what to do for the positive case’s family members and the necessity of closing our company. At this time, there are no employees who are suspected of being infected*” (ID = 2).

#### 3.2.2. Extreme Anxiety and Fear of COVID-19

The consulters showed extreme anxiety, fear, and concerns regarding COVID-19 because it is an unknown infectious disease. Additionally, they complained about the administrative system’s response to COVID-19 and unreasonably reprimanded the PHNs.

“(*I do not have any COVID-19 symptoms now.) However, I want to know the concrete symptom of breathlessness because I am concerned about it*.” (ID = 154).

#### 3.2.3. Opinions/Inquiries Regarding Healthcare and Medical Systems for Responding to COVID-19

Due to the limited capacity of PCR testing and designated medical hospitals that could accept inpatients with COVID-19, the callers asked the healthcare and medical systems to respond to their concerns about COVID-19. For example, they asked about the rationale and structure of PCR testing, the COVID-19 measures introduced by the national or local government and PHCs, and the medical system.

“*I want to know about the medical system of City A regarding COVID-19.*” (ID = 105).

“*Is it free of charge regarding the COVID-19-related medical fee? Who would pay for the patient’s transport to the hospital*?” (ID = 27).

#### 3.2.4. Discrimination and Misunderstandings Due to Lack of Proper Knowledge about COVID-19

Lack of proper knowledge about COVID-19 led to complaints and concerns about being in close contact with COVID-19 cases. Additionally, this category included complaints about discriminatory behaviors and harassment, such as discriminatory remarks about medical professionals, patients, and others trying to crack down on people who not following COVID-19-related infection control rules in the workplace. Moreover, in Japan, as medical institutions are typically designated as either facilities that deal with infectious diseases or “general hospitals” (which do not normally treat infectious diseases), the staff of general hospitals did not know how to correctly respond to COVID-19 in the first wave of the pandemic. Therefore, PHNs were asked to refrain from providing consultations at those medical facilities, and some physicians did not treat COVID-19 patients.

“*A returnee from overseas who should stay at home was out. I want you to crack down on them.*” (ID = 159).

“*I think the person would be positive COVID-19 based on the symptoms that I heard on the phone. However, if I saw the patient like that, I would get infected with COVID-19*.” (ID = 165).

#### 3.2.5. Consultations Regarding COVID-19 Outbreaks within an Organization

This category included the only call received from a caller working at an organization. It included the topic of business continuity when a new COVID-19 case was detected in a healthcare or medical facility and how to respond to it as an organization.

“*I heard that an employee of another company whom I had a meeting with the other day was COVID-19 positive. I want to know what we should do first as a company*.” (ID = 73).

#### 3.2.6. Consultations Regarding Infection Prevention Measures for COVID-19

There were consultations regarding infection prevention measures in various situations, including for individuals and groups (e.g., companies, schools, social welfare facilities), in daily life, for overseas returnees, and in cases of contact with COVID-19 cases or suspected cases.

“*Is it okay to let my child play in the park? If yes, what should we be careful about*?” (ID = 175).

“*A person returning from overseas is staying at my hotel. How can we disinfect the hotel after they check out*?” (ID = 160).

#### 3.2.7. Inquiries about the Infection Status of City A

There were also consultations regarding providing information to understand City A’s infection situation, including questions about the total number of new COVID-19 cases in City A and free telephone consultations in City A.

“*I would like to know the number and content of telephone consultations received by City A*.” (ID = 188).

### 3.3. Categorization of Consultations According to Whether PHNs Needed to Respond to It or Not

**Figure 1:**
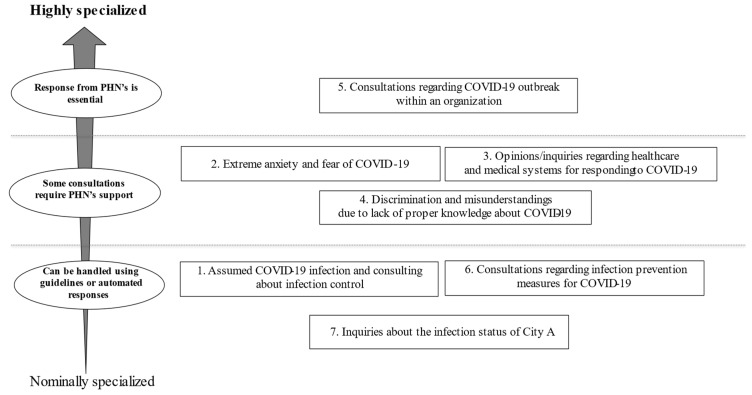
The categorization according to whether the PHNs needed to respond to the call.

Figure 1 shows the categorization according to whether the PHNs needed to respond to the call. There were three categories for which PHNs would not be required to respond if the information was made available in a manual (as manuals for responding to concerns had not been developed or were not adequate early in the pandemic). These categories are “Assumed COVID-19 infection and consulting about infection control”, “Consultations regarding infection prevention measures for COVID-19”, and “Inquiries about the infection status of City A”.

Additionally, another three categories did not need to be handled by PHNs once a manual was developed: “Emotional labor” referring, to regulating or managing emotional aspects of work during the pandemic; “Discrimination and misunderstandings due to lack of proper knowledge about COVID-19”; and “the need for healthcare and medical systems information about COVID-19 to provide correct information”. However, if some consultants were not satisfied with the answers in the manual, they would have needed to respond to them.

Consultations regarding a COVID-19 outbreak within an organization need to be responded to by PHNs. Thus, a high level of expertise is required to deal with this category.

## 4. Discussion

This cross-sectional study analyzed unclassified PHC telephone consultations during the first wave of COVID-19 and classified them into seven categories. The most common topic was people who assumed that they had or would be exposed to (or have) COVID-19 and wanted to speak to someone about infection control. People within the community also consulted with PHNs in anticipation of becoming infected with COVID-19 in the future. However, the telephone consultation service was initially intended for returnees from abroad or those suspected of coming into contact with COVID-19 patients who needed to be in isolation or who had a fever and respiratory symptoms to encourage them to contact PHCs to obtain the information they needed [16]. The results suggest that the first telephone consultation system could not handle all the types of general questions asked by residents regarding COVID-19, and the inadequacy of the system exacerbated the busyness of PHNs.

The results showed that the second most common topic of these conversations was people’s extreme anxiety and fear of COVID-19. This finding is in line with the results of previous studies, showing that people in the community felt anxiety and panic early on in the COVID-19 pandemic [21,22], as well as a meta-analysis that showed that telemedicine is a helpful tool for providing psychological support to prevent COVID-19 infection [23]. In Japan, mental health and welfare centers and psychiatric institutions provided supportive listening to COVID-19 patients, their family members, and the general public [24]. However, these facilities did not provide adequate factual knowledge or platforms to help reduce people’s anxiety and fear about COVID-19. Thus, it is likely that the PHC telephone consultation service reduced anticipatory anxiety and fear of COVID-19 by responding to the needs of individuals.

These results also showed that consultations included questions about healthcare and medical systems’ responses to COVID-19. As COVID-19 was an unknown infectious disease, PHCs tried to provide sustainable health services while controlling the spread of COVID-19 for people in the community by implementing traditional infection control strategies, including contact tracing and the early detection of latent cases [9]. However, the use of these strategies was not shared with the general public. Therefore, PHCs and PHNs need to share their infection control strategies with residents during ordinary times in order to build community understanding.

The unclassified consultations included instances of discrimination and misunderstandings about COVID-19. Although PHNs and PHC staff have played crucial roles in health consultations about COVID-19, they also had to listen to complaints regarding the administrative system and endure unreasonable reprimands. These people had their own physical and mental health burdens and insomnia and were at high risk of psychological distress and depression due to the lack of adequate support [25]. Unreasonable and illegitimate work is associated with emotional exhaustion and negative feelings related to working [26]. Additionally, the PHC revealed detailed information about COVID-19 cases, which triggered the problem of COVID-19 stigma during the first wave of the COVID-19 pandemic [27]. Hence, each local government should reconsider risk communication regarding infectious disease outbreaks and develop support systems for PHC staff to help maintain their physical and mental health as soon as possible.

We found that questions about responses to the COVID-19 outbreak were only received from people working in organizations. In Japan, identifying positive COVID-19 cases and conducting contact tracing were prioritized in order to prevent the development of new clusters [28]. Furthermore, the primary strategies used for a population strategy are modifying public behavior and adopting personal protection measures, including wearing a mask with respiratory etiquette, maintaining physical distance measures, and maintaining hand hygiene [29,30]. However, the response to organizations with COVID-19 cases was not adequately developed except in clusters. For this reason, organizations with COVID-19 cases had no choice but to use the telephone counseling service for people in the community suspected of having COVID-19 symptoms. To prevent a new cluster, PHNs should handle this type of consultation preferentially and prepare to collaborate with organizations.

The results showed that consultations were about a mixture of things that PHNs should handle and things they should not, such as implementing COVID-19 prevention measures and measures for dealing with the infection status of City A. Sharing and shifting tasks are crucial in order to provide sustainable essential health services [11]. Due to the rapidly increasing number of COVID-19 cases and their busy schedules, PHNs face difficulties in focusing on infection control routines [31]. In addition, the total number of PHNs was limited. Thus, the directors of PHNs and PHCs should consider that PHNs should only deal with essential consultations and develop a manual and a list of frequent questions and answers for outsourcing tasks requiring a low level of skill or expertise in infection control for responding by workers in the call center. They could also consider using an artificial intelligence-programmed consultation web-based system. PHNs should focus on providing consultations on COVID-19 outbreaks in organization, which require a high level of expertise and skills in infection control.

This study has several limitations. First, this cross-sectional study could not identify a causal relationship using limited data from one city. Second, we analyzed unclassified consultation data obtained in a previous study [17] and did not perform a power analysis in this study. Thus, we could not generalize the results to other local governments in Japan. Third, due to continuation and worsening of the COVID-19 pandemic, we could not confirm the results obtained for the PHNs in City A.

## 5. Conclusions

This cross-sectional study analyzed unclassified PHC telephone consultations during the first wave of the COVID-19 pandemic. The most common consultation topics was assumed COVID-19 infection and infection control; the second was extreme anxiety and fear of COVID-19. Other consultations were a mixture of things that PHNs should handle and things that they should not. Each local government and PHC should consider sharing and task-shifting telephone consultation services among PHNs and other staff to reduce the burden on PHNs and allow them to concentrate on conducting infection control more effectively.

## Figures and Tables

**Table 1:** Characteristics of COVID-19-related free telephone consultation cases (*n* = 188).

Variables	Total *n* (%) or Mean (SD)
Classification of consultor	
Residents	99 (52.7)
Organizations	89 (47.3)
Sex	
Male	115 (61.2)
Age (*n* = 80)	
Under 20 years old	4 (5.0)
20–29	13 (16.3)
30–39	19 (23.8)
40–49	12 (15.0)
50–59	6 (7.5)
60–69	10 (12.5)
70–79	10 (12.5)
80 years old and over	6 (7.5)
Duration of consultation: minutes (*n* = 135)	7.0 (4.6)

Note: SD: standard deviation. The “organizations” group included the staff of companies, administrative organs, educational institutions, and health and welfare-related organizations in City A.

**Table 2:** Categories of the 207 “other” consultation items.

Category	Subcategory	*n*
1. Assumed COVID-19 infection and consulting about infection control (62)	The initial response measure in case of a COVID-19 outbreak (including suspected cases).	46
	How to communicate to organizational members if a COVID-19 outbreak appears in the organization.	7
	Criteria for restrictions and self-isolation for COVID-19 cases (including suspected cases).	6
	Continuity of care when a caregiver is infected with COVID-19 or is otherwise unable to provide care.	3
2. Extreme anxiety and fear of COVID-19 (50)	Concern about contact with the person with COVID-19.	20
	Fear of COVID-19 infection with apparent reason.	17
	Fear of COVID-19 infection without apparent reason.	5
	Complaints regarding the administrative system or unreasonable reprimands.	6
	Vague anxiety associated with the expansion of the COVID-19 pandemic.	2
3. Opinions/inquiries regarding healthcare and medical systems for responding to COVID-19 (30)	The rationale and structure of conducting PCR testing.	17
	COVID-19 measures introduced by the national or local government and PHCs.	16
	Medical systems used for responding to COVID-19 in City A.	7
4. Discrimination and misunderstandings due to lack of proper knowledge about COVID-19 (24)	Trying to crack down on people who do not follow the infection control rules.	12
	Harassment regarding COVID-19 in the workplace.	4
	Refusing a consultation at the hospital or clinic.	5
	Refusal of medical treatment by physicians.	3
5. Consultations regarding COVID-19 outbreak within an organization (18)	Business continuity when a COVID-19 case occurs (including suspected infections) in healthcare or medical facilities.	12
	The initial response of the organization when the COVID-19 case occurred (including suspected infections).	6
6. Consultations regarding infection prevention measures for COVID-19 (15)	Infection prevention measures for community groups (e.g., companies, schools, social welfare facilities).	6
	Infection prevention measures for daily life.	4
	Infection prevention measures for overseas returnees.	3
	Infection prevention measures when someone has been in contact with COVID-19 patients or someone suspected to have COVID-19.	2
7. Inquiries about the infection status of City A (8)	Number of new COVID-19 cases in City A.	6
	Number of free telephone consultations in City A.	2

Note. *n*: total number of units; PCR: polymerized chain reaction; PHCs: public health centers.

## Data Availability

The data presented in this study are not publicly available because of privacy restrictions.
